# Association between weekend warrior physical activity pattern and all-cause mortality among adults living with type 2 diabetes: a prospective cohort study from NHANES 2007 to 2018

**DOI:** 10.1186/s13098-024-01455-0

**Published:** 2024-09-12

**Authors:** Jinli Mahe, Ao Xu, Li Liu, Lei Hua, Huiming Tu, Yujia Huo, Weiyuan Huang, Xinru Liu, Jian Wang, Jinhao Tang, Yang Zhao, Zhining Liu, Qiaojun Hong, Rong Ye, Panpan Hu, Peng Jia, Junjie Huang, Xiangyi Kong, Zongyuan Ge, Aimin Xu, Longfei Wu, Chaopin Du, Feng Shi, Hanbin Cui, Shengfeng Wang, Zhihui Li, Liang Wang, Lei Zhang, Lin Zhang

**Affiliations:** 1 The School of Public Health and Preventive Medicine, Monash University, Melbourne, Australia; 2 Suzhou Industrial Park Monash Research Institute of Science and Technology, Monash University, Suzhou, China; 3 Monash University-Southeast University Joint Research Institute (Suzhou), Southeast University, Suzhou, China; 4 Data Centre, Affiliated Hospital of Jiangnan University, Wuxi, China; 5 Department of Gastroenterology, Affiliated Hospital of Jiangnan University, Wuxi, China; 6 International College, Krirk University, Bangkok, Thailand; 7 The George Institute for Global Health, University of New South Wales, Sydney, Australia; 8 The George Institute for Global Health, Beijing, China; 9 Anhui Medical University Affiliated Hospital, Hefei, China; 10 Anhui No.2 Provincial People’s Hospital, Hefei, China; 11 The School of Mental Health and Psychological Sciences, Anhui Medical University, Hefei, China; 12 Anhui Province Key Laboratory of Cognition and Neuropsychiatric Disorders, Hefei, China; 13 Collaborative Innovation Canter of Neuropsychiatric Disorders and Mental Health, Hefei, China; 14 Department of Neurology, the First Affiliated Hospital of Anhui Medical University, Hefei, China; 15 School of Resource and Environmental Sciences, Wuhan University, Wuhan, China; 16 Hubei Luojia Laboratory, Wuhan, China; 17 School of Public Health, Wuhan University, Wuhan, China; 18 International Institute of Spatial Lifecourse Health (ISLE), Wuhan University, Wuhan, China; 19 The Jockey Club School of Public Health and Primary Care, Faculty of Medicine, Chinese University of Hong Kong, Hongkong, China; 20 Department of Breast Surgical Oncology, National Cancer Centre/National Clinical Research Centre for Cancer/Cancer Hospital, Chinese Academy of Medical Sciences and Peking Union Medical College, Beijing, China; 21 Faculty of Engineering, Monash University, Melbourne, Australia; 22 College of Big Data and Software Engineering, Zhejiang Wanli University, Ningbo, China; 23 Centre for Genetic Epidemiology and Genomics, School of Public Health, Jiangsu Key Laboratory of Preventive and Translational Medicine for Geriatric Diseases, MOE Key Laboratory of Geriatric Diseases and Immunology, Suzhou Medical College of Soochow University, Suzhou, China; 24 Department of Neurology, Nantong Third Hospital Affiliated to Nantong University, Nantong, China; 25 Department of Surgery, Civil Aviation General Hospital, Shanghai, China; 26 Department of Cardiovascular Medicine, First Affiliated Hospital, Ningbo University, Ningbo, China; 27 School of Public Health, Peking University, Beijing, China; 28 Tsinghua Vanke School of Public Health, Beijing, China; 29 Department of Public Health, Marshall University, Huntington, WV USA; 30 Central Clinical School, Faculty of Medicine, Nursing and Health Sciences, Monash University, Melbourne, Australia; 31 Clinical Medical Research Centre, Children’s Hospital of Nanjing Medical University, Nanjing, China; 32 Melbourne Sexual Health Centre, Alfred Health, Melbourne, Australia

**Keywords:** Physical activity, Weekend warrior, Regularly active, All-cause mortality, Type 2 diabetes

## Abstract

**Background:**

It is uncertain whether the weekend warrior pattern is associated with all-cause mortality among adults living with type 2 diabetes. This study explored how the ‘weekend warrior’ physical activity (PA) pattern was associated with all-cause mortality among adults living with type 2 diabetes.

**Methods:**

This prospective cohort study investigated US adults living with type 2 diabetes in the National Health and Nutrition Examination Survey (NHANES). Mortality data was linked to the National Death Index. Based on self-reported leisure-time and occupational moderate-to-vigorous PA (MVPA), participants were categorized into 3 groups: physically inactive (< 150 min/week of MVPA), weekend warrior (≥ 150 min/week of MVPA in 1 or 2 sessions), and physically active (≥ 150 min/week of MVPA in 3 or more sessions).

**Results:**

A total of 6067 participants living with type 2 diabetes [mean (SD) age, 61.4 (13.5) years; 48.0% females] were followed for a median of 6.1 years, during which 1206 deaths were recorded. Of leisure-time and occupational activity, compared with inactive individuals, hazard ratios (HRs) for all-cause mortality were 0.49 (95% CI 0.26–0.91) and 0.57 (95% CI 0.38–0.85) for weekend warrior individuals, and 0.55 (95% CI 0.45–0.67) and 0.64 (95% CI 0.53–0.76) for regularly active individuals, respectively. However, when compared leisure-time and occupational weekend warrior with regularly active participants, the HRs were 0.82 (95% CI 0.42–1.61) and 1.00 (95% CI 0.64–1.56) for all-cause mortality, respectively.

**Conclusions:**

Weekend warrior PA pattern may have similar effects on lowering all-cause mortality as regularly active pattern among adults living with type 2 diabetes, regardless of leisure-time or occupational activity. Therefore, weekend warrior PA pattern may be sufficient to reduce all-cause mortality for adults living with type 2 diabetes.

**Supplementary Information:**

The online version contains supplementary material available at 10.1186/s13098-024-01455-0.

## Summary box

### What is already known on this topic?

- Despite meta-analyses and observational studies showed that the leisure-time ‘weekend warrior’ physical activity pattern reduced all-cause mortality in the general population, critics argue that this pattern was not sufficient to lower the risk of all-cause mortality in adults living with type 2 diabetes, who engaged in less PA than those without chronic diseases.
- Moreover, it remains unknown whether different types of physical activity had different effects on all-cause mortality among patients living with type 2 diabetes.

### What this study adds?

- The study indicated that the ‘weekend warrior’ had similar effects on lowering all-cause mortality as regularly active pattern among adults living with type 2 diabetes, regardless of leisure-time or occupational physical activity.
- The weekend warrior PA pattern may be sufficient to reduce all-cause mortality for adults living with type 2 diabetes.

### How this study might affect research, practice or policy?

- Our findings serve to motivate inactive adults living with type 2 diabetes to engage in at least 150 min per week of moderate-to-vigorous physical activity, achievable through daily leisure-time and occupational physical activity.

## Introduction

In 2021, the International Diabetes Federation reported 537 million cases of diabetes globally, projected to increase to 783 million by 2045 [1]. In the United States, approximately 32 million adults had diabetes, expected to rise to 36 million by 2045 [2]. Over 90% of these cases are attributed to type 2 diabetes [3]. Globally, 6.7 million deaths were attributed to diabetes, with a mortality rate of 32.6% related to diabetes among individuals under the age of 60 [1].

Physical activity (PA) has been promoted as the primary measures to prevent numerous chronic complications associated with type 2 diabetes [4]. Higher PA levels also reduce mortality risk in individuals living with type 2 diabetes [5]. The 2020 World Health Organization guidelines recommend that adults engage in 150 to 300 min per week (min/week) of moderate-intensity PA (MPA), 75 to 150 min/week of vigorous-intensity PA (VPA), or equivalent combinations [6]. Nevertheless, limited studies in high-income countries indicated that the adherence rate to these guidelines among adults living with type 2 diabetes was only 40%–60% [7]. Therefore, the transition of inactive adults living with type 2 diabetes from inactive lifestyles to compliance with minimum PA guidelines could result in favorable health outcomes.

Currently, the PA frequency has not be clearly defined. Individuals who focus all their recommended moderate-to-vigorous PA (MVPA) within 1 or 2 weekly sessions are referred to as ‘weekend warriors’, whereas those engaging in 3 or more sessions are classified as regularly active participants [8]. A meta-analysis suggested that weekend warrior and regularly active PA patterns had similar benefits in lowering all-cause and cardiovascular disease (CVD) mortality among the general population [9]. Nevertheless, it remains uncertain whether both the "weekend warrior" and consistently active patterns of PA yield similar benefits in mitigating mortality risk among adults living with type 2 diabetes. Additionally, above research only focused on leisure-time PA but neglected occupational PA which accounts for an important part in daily PA. A study found both leisure-time and occupational PA patterns provided similar effect on decreasing all-cause and CVD mortality among adults living with type 2 diabetes [10]. On the contrary, another study showed that participants in higher occupational PA seemed to have higher risk for all-cause mortality in CVD patients [11]. Thus, limited and contradictory studies could not conclude whether different types of PA have different benefit in all-cause mortality in patients living with type 2 diabetes. It also remains uncertain whether the effect of weekend warrior PA pattern, compared with physical inactivity, on mortality differs based on frequency, duration and intensity among adults living with type 2 diabetes.

In summary, for adults living with type 2 diabetes, this study investigated whether the weekend warrior pattern of leisure-time and occupational activity had a similar impact on all-cause mortality as the regular activity pattern, and also estimated the potential effects of the frequency, duration, and intensity of PA on all-cause mortality.

## Methods

### Study population

The National Center for Health Statistics (NCHS) Ethics Review Board approved the research protocols of the National Health and Nutrition Examination Survey (NHANES), and all participants provided written informed consent. Details of the NHANES have been described in previous research [12]. Our study included six cycles of NHANES data from 2007–2008 to 2017–2018, focusing on individuals aged 18 years or older living with type 2 diabetes. Type 2 diabetes was diagnosed based on self-reporting, fasting plasma glucose levels ≥ 7.0 mmol/L, or plasma HbA1c levels ≥ 6.5% (or 48 mmol/mol) [13]. Out of 59,842 adults, 6089 were diagnosed with type 2 diabetes. We excluded pregnant women (n = 7), individuals without PA records (n = 4), and probable subjects living with type 1 diabetes (defined as those under 20 years old who were only using insulin) (n = 11) [14]. Ultimately, the analysis included 6,067 participants living with type 2 diabetes (Additional file 1: Fig S2).

### Evaluation of PA

PA was assessed using the Global Physical Activity Questionnaire [12]. Frequency and duration of sessions were collected according to 8 questions (Additional file 1: Table S1). Leisure-time PA was defined as sports, fitness, or recreational activities. Occupational PA included work and chore-related activities. MPA and VPA were characterized by relatively small and large increases in breathing or heart rate, respectively. According to PA guidelines, 1 min of VPA was considered equivalent to 2 min of MPA [15]. Leisure-time and occupational MVPA was calculated by multiplying frequency and duration of sessions. Therefore, the quantification of MVPA (min/week) was calculated with the formula: 2 × VPA + MPA. The amount of total MVPA were calculated by summing leisure-time and occupational MVPA minutes. A detailed example was shown in Additional file 1: Methods-S1.

Of leisure-time, occupational and total PA, participants were categorized into 3 groups: inactive, accumulating less than 150 min/week of MVPA; weekend warrior, accumulating at least 150 min/week of MVPA in 1 or 2 sessions; physically active, accumulating at least 150 min/week of MVPA in 3 or more sessions. Lastly, for leisure-time, occupational and total PA, the weekend warrior and regularly active groups were further categorized according to duration in each session (calculated as MVPA minutes divided by weekly sessions) and intensity (calculated as the VPA divided by MVPA). The MVPA duration in each session and intensity levels were defined as: ≤ 30 or > 30 min/session, ≤ 30% or > 30%, respectively. A detailed example was shown in Additional file 1: Methods-S2.

### Mortality ascertainment

To ascertain mortality data in the study population, the NHANES public-use linked mortality file through December 31, 2019, incorporating a distinct study identifier, was employed [16]. The data linkage between this file and the National Death Index was conducted by the NCHS using a probability matching algorithm. Additional information regarding the specific matching methodology can be obtained directly from the NCHS [17].

### Covariates

Information on various covariates, including age, gender, race and ethnicity, levels of education and income, marital status, cigarette smoking, alcohol intake, and diet quality were obtained through standardized questionnaires. Educational and income levels, marital status, cigarette smoking, and alcohol intake were each categorized into 3 groups. According to federal poverty income ratio (PIR), income levels were classified into 3 levels. Healthy eating index-2015 (HEI-2015) was used to evaluate diet quality [18], then divided into tertile. Body mass index (BMI) was calculated as weight (kg) divided by the square of height (m), then categorized into 4 levels. To consider participants’ heath condition, self-reported diagnoses of hypertension and high cholesterol, were also considered. Familial diabetes was assessed using the question, “Including living and deceased, were any of your close biological that is, blood relatives including father, mother, sisters or brothers, ever told by a health professional that they had diabetes?”.

### Handling of missing variables

Additional file 1: Table S2 showed the distribution of missing variables among adults living with type 2 diabetes in this study. Multiple imputation method was employed to maintain the largest possible sample size.

### Statistical analysis

Although subsample weights are provided for each group, the NCHS advises against using them because combining subsamples can reduce the sample size, leading to unstable and unreliable statistical estimates [19, 20]. Therefore, the associations of PA patterns with all-cause and cause-specific mortality rates were assessed using unweighted Cox proportional hazards regression models, with calendar time (months) as the time variable. Hazard ratios (HRs) and 95% CIs were calculated, and covariates adjusted in models were selected according to previous studies [21, 22]. Model 1 was adjusted for age, gender, race and ethnicity. Model 2 was further adjusted for educational attainment, income, marital status, smoking, alcohol intake, HEI-2015, high blood cholesterol, family diabetes, and hypertension, then Model 3 was further adjusted for BMI based on Model 2. The possibility of type I error may have been elevated because of multiple comparisons, and as a result, caution is warranted when interpreting the findings. To evaluate interaction incorporating the cross-terms of PA patterns and gender, the adjusted Wald test was used. Given the absence of evidence for interaction by gender, the results were presented for the whole participant sample.

Two sensitivity analyses were performed to assess the robustness of our findings in adults living with type 2 diabetes. In the first sensitivity analysis, of leisure-time and occupational PA, individuals engaging in ≥ 600 min/week of PA were excluded in order to address variability of different exposure and improve comparability between weekend warriors and regularly active participants. In the second sensitivity analysis, individuals who passed away within the first 24 months of follow-up were excluded to mitigate potential confounding from preexisting diseases. The statistical analysis was completed in January 2024 using SPSS software (version 26; IBM Corp), with all tests being two-tailed and statistical significance defined as *P* < 0.05.

## Results

### Baseline characteristics of participants

A total of 6067 participants [mean (SD) age, 61.4 (13.5) years; 2911 (48.0%) females; 1740 (28.7%) Hispanic, 2020 (33.3%) non-Hispanic White, and 1600 (26.4%) non-Hispanic Black individuals] living with type 2 diabetes were included. During a median follow-up of 6.1 years (37,008 person-years), 1206 deaths were documented. For leisure-time PA, 4885 (80.5%) were inactive, 128 (2.1%) were weekend warriors, and 1054 (17.4%) were regularly active participants. Regularly active group tended to be younger, males, leaner, current drinkers, and had higher educational levels, income, and HEI-scores, as well as a lower prevalence of high blood cholesterol and hypertension compared with physically inactive group. Compared with regularly active participants, weekend warriors tended to be younger, males, Hispanic, married or living with partner, overweight or obese, former and current smokers, current drinkers, and had higher levels of education and income (Table 1). For leisure-time PA, weekend warrior group had a median PA of 290 min/week, and regularly active group had 470 min/week. These results were similar in occupational PA (Additional file 1: Table S3).

**Table 1:** Baseline characteristics of participants in leisure-time PA pattern

Variable	Leisure-time PA pattern, No. (%)			Overall
	Inactive	Weekend warrior	Regularly active	
Overall	4885 (80.5)	128 (2.1)	1054 (17.4)	6067 (100.0)
Age				
18–44	538 (11.0)	30 (23.4)	177 (16.8)	745 (12.3)
45–64	2089 (42.8)	51 (39.8)	478 (45.4)	2618 (43.2)
65–84	2258 (46.2)	47 (36.7)	399 (37.9)	2704 (44.6)
Gender				
Male	2402 (49.2)	97 (75.8)	657 (62.3)	3156 (52.0)
Female	2483 (50.8)	31 (24.2)	397 (37.7)	2911 (48.0)
Race and ethnicity				
Hispanic	1407 (28.8)	44 (34.4)	289 (27.4)	1740 (28.7)
Non-Hispanic White	1669 (34.2)	45 (35.2)	306 (29.0)	2020 (33.3)
Non-Hispanic Black	1285 (26.3)	28 (21.9)	287 (27.2)	1600 (26.4)
Other	524 (10.7)	11 (8.6)	172 (16.3)	707 (11.7)
Education level				
< High school degree	1854 (38.0)	23 (18.0)	235 (22.3)	2112 (34.8)
High school degree	1146 (23.5)	23 (18.0)	228 (21.6)	1397 (23.0)
> High school degree	1885 (38.6)	82 (64.1)	591 (56.1)	2558 (42.2)
Poverty income ratio				
< 1	1123 (23.0)	16 (12.5)	178 (16.9)	1317 (21.7)
[1,3)	2618 (53.6)	61 (47.7)	494 (46.9)	3173 (52.3)
≥ 3	1144 (23.4)	51 (39.8)	382 (36.2)	1577 (26.0)
Marital status				
Married/living with partner	2826 (57.9)	92 (71.9)	641 (60.8)	3559 (58.7)
Widowed/divorced/separated	1602 (32.8)	23 (18.0)	291 (27.6)	1916 (31.6)
Never married	457 (9.4)	13 (10.2)	122 (11.6)	592 (9.8)
BMI				
≤ 18.4	20 (0.4)	1 (0.8)	1 (0.1)	22 (0.4)
18.5–24.9	583 (11.9)	10 (7.8)	155 (14.7)	748 (12.3)
25–29.9	1259 (25.8)	44 (34.4)	342 (32.4)	1645 (27.1)
≥ 30	3023 (61.9)	73 (57.0)	556 (52.8)	3652 (60.2)
Cigarette smoking status				
Never	2451 (50.2)	61 (47.7)	568 (53.9)	3080 (50.8)
Former	1743 (35.7)	51 (39.8)	385 (36.5)	2179 (35.9)
Current	691 (14.1)	16 (12.5)	101 (9.6)	808 (13.3)
Alcohol intake				
Never	2261 (46.3)	41 (32.0)	410 (38.9)	2712 (44.7)
Former	1293 (26.5)	23 (18.0)	233 (22.1)	1549 (25.5)
Current	1331 (27.2)	64 (50.0)	411 (39.0)	1806 (29.8)
HEI-2015				
T1	1687 (34.5)	57 (44.5)	279 (26.5)	2023 (33.3)
T2	1660 (34.0)	33 (25.8)	329 (31.2)	2022 (33.3)
T3	1538 (31.5)	38 (29.7)	446 (42.3)	2022 (33.3)
High blood cholesterol				
Yes	2915 (59.7)	72 (56.3)	590 (56.0)	3577 (59.0)
No	1970 (40.3)	56 (43.8)	464 (44.0)	2490 (41.0)
Family diabetes				
Yes	3223 (66.0)	91 (71.1)	714 (67.7)	4028 (66.4)
No	1662 (34.0)	37 (28.9)	340 (32.3)	2039 (33.6)
Hypertension				
Yes	3312 (67.8)	74 (57.8)	639 (60.6)	4025 (66.3)
No	1573 (32.2)	54 (42.2)	415 (39.4)	2042 (33.7)

BMI, body mass index; HEI-2015, healthy eating index-2015

### Association between PA patterns and mortality

For leisure-time, occupational and total PA, compared with physically inactive participants, the HRs for all-cause mortality were 0.49 (95% CI 0.26–0.91), 0.57 (95% CI 0.38–0.85) and 0.60 (95% CI 0.41–0.90) for weekend warrior, and 0.55 (95% CI 0.45–0.67), 0.64 (95% CI 0.53–0.76) and 0.56 (95% CI 0.49–0.65) for regularly active participants (Table 2). In weekend warrior and regularly active patterns, lower all-cause mortality was also observed from Kaplan–Meier curves (Additional file 1: Figs. S3–S4). For cause-specific mortality rates, in leisure-time PA, when comparing with physically inactive individuals, only regularly active pattern was associated with lower CVD mortality risk. Likewise, in occupational PA, regularly active subjects had lower CVD and cancer mortality risk than inactive subjects (Additional file 1: Table S4).

**Table 2:** Association between PA patterns and all-cause mortality

Variable	Deaths	Participants	Mortality, HR (95% CI)		
			Model 1^a^	Model 2^b^	Model 3^c^
Leisure-time					
Inactive	1087	4885	1 [Reference]	1 [Reference]	1 [Reference]
Weekend warrior	10	128	0.37 (0.20–0.70)	0.49 (0.26–0.91)	0.49 (0.26–0.91)
Regularly active	109	1054	0.49 (0.40–0.60)	0.56 (0.46–0.68)	0.55 (0.45–0.67)
Occupational					
Inactive	1029	4570	1 [Reference]	1 [Reference]	1 [Reference]
Weekend warrior	24	150	0.53 (0.35–0.79)	0.56 (0.38–0.85)	0.57 (0.38–0.85)
Regularly active	153	1347	0.57 (0.48–0.68)	0.63 (0.53–0.75)	0.64 (0.53–0.76)
Total					
Inactive	934	3759	1 [Reference]	1 [Reference]	1 [Reference]
Weekend warrior	25	155	0.56 (0.38–0.84)	0.60 (0.41–0.90)	0.60 (0.41–0.90)
Regularly active	247	2153	0.50 (0.43–0.58)	0.56 (0.49–0.65)	0.56 (0.49–0.65)

^a^Model 1 Adjusted for age, gender, race and ethnicity

^b^Model 2 Adjusted for Model 1 and plus education, income, marital status, smoking, alcohol intake, HEI-2015, high blood cholesterol, family diabetes, hypertension

^c^Model 3 Adjusted for Model 2 and BMI

### Association of weekend warrior and regularly active patterns with mortality

**Fig. 1:**
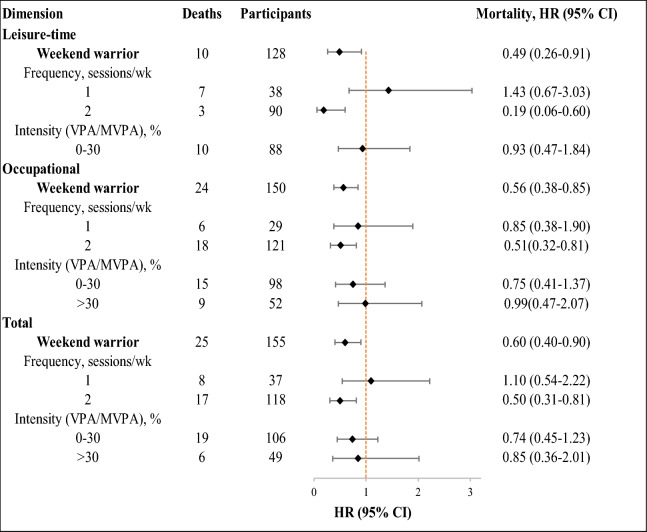
Association between weekend warrior PA pattern and all-cause mortality, by frequency and intensity of activity. Estimates were from the fully adjusted model that included the covariates of age, gender, race and ethnicity, education, income, marital status, smoking, alcohol intake, HEI-2015, high blood cholesterol, family diabetes, hypertension, and BMI

**Fig. 2:**
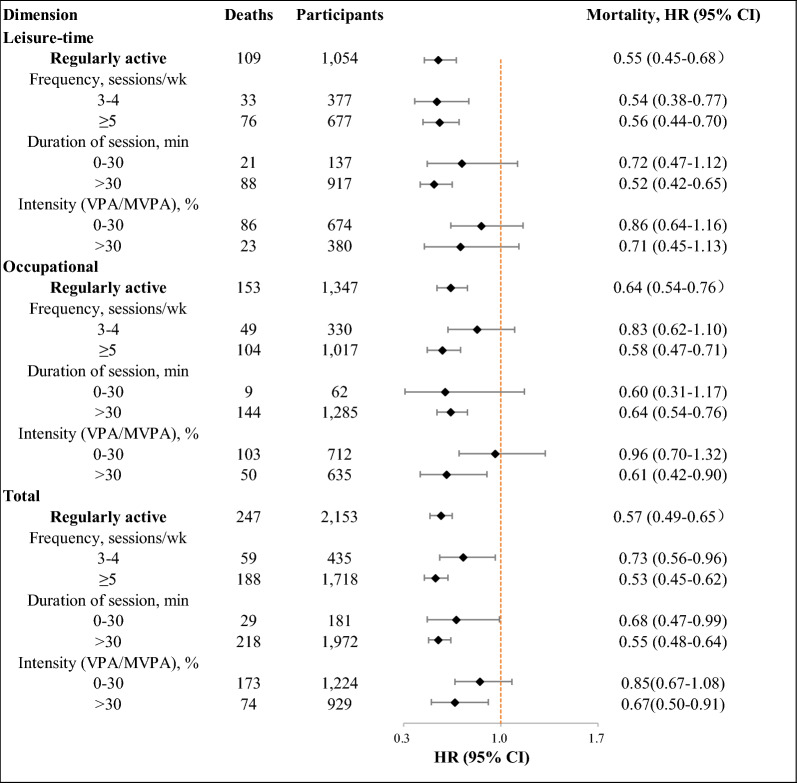
Association between regularly active PA pattern and all-cause mortality, by frequency, duration, and intensity of activity. Estimates were from the fully adjusted model that included the covariates of age, gender, race and ethnicity, education, income, marital status, smoking, alcohol intake, HEI-2015, high blood cholesterol, family diabetes, hypertension, and BMI

Of leisure-time, occupational and total PA, when compared with inactive pattern, weekend warrior pattern with higher frequency were associated with lower mortality. HRs for all-cause mortality were 0.19 (95% CI 0.06–0.60), 0.51 (95% CI 0.32–0.81) and 0.50 (95% CI 0.31–0.81) for 2 sessions/week, respectively. However, no association between intensity of PA and mortality was found (Fig. 1 and Additional file 1: Table S5). In leisure-time PA, participants in physically active group with both frequencies had lower mortality compared with those in inactive group. The HRs were 0.54 (95% CI 0.38–0.77) and 0.56 (95% CI 0.44–0.70). Association between longer duration of session and lower mortality was found, and the HR was 0.52 (95% CI, 0.42–0.65). However, association between intensity and mortality was not observed. Of occupational PA, when compared regularly active group with inactive group, higher frequency, duration of session and intensity were associated with lower mortality risk. The HRs were 0.58 (95% CI 0.47–0.71), 0.64 (95% CI 0.54–0.76) and 0.61 (95% CI 0.42–0.90). Similarly, for total PA, higher frequency, duration of session and intensity of physically active pattern also provided protection against all-cause mortality compared with inactive group (Fig. 2 and Additional file 1: Table S6).

### Association between weekend warrior PA pattern and mortality

Of leisure-time, occupational and total PA, compared with regularly active participants, the HRs for weekend warrior participants were 0.82 (95% CI 0.42–1.61), 1.00 (95% CI 0.64–1.56) and 1.09 (95% CI 0.71–1.65) for all-cause mortality. Consistently, no associations with all-cause mortality were found regardless of the frequency and intensity of PA (Table 3). The results were similar for CVD and cancer mortality rates (Additional file 1: Tables S7–S8).

**Table 3:** Association between weekend warrior PA pattern and all-cause mortality, with regularly active pattern as the reference group

Dimension	Deaths	Participants	Mortality, HR (95% CI)		
			Model 1^a^	Model 2^b^	Model 3^c^
Leisure-time					
Regularly active	109	1054	1 [Reference]	1 [Reference]	1 [Reference]
Weekend warrior	10	128	0.73 (0.38–1.40)	0.83 (0.42–1.62)	0.82 (0.42–1.61)
Frequency, sessions/week					
1	7	38	1.84 (0.85–4.01)	2.13 (0.93–4.89)	2.11 (0.92–4.85)
2	3	90	0.33 (0.10–1.10)	0.35 (0.11–1.11)	0.35 (0.11–1.11)
Intensity (VPA/MVPA), %					
0–30	10	88	0.90 (0.47–1.73)	1.01 (0.52–1.99)	1.01 (0.51–1.97)
> 30	0	40	NA	NA	NA
Occupational					
Regularly active	153	1347	1 [Reference]	1 [Reference]	1 [Reference]
Weekend warrior	24	150	0.99 (0.64–1.53)	1.02 (0.66–1.60)	1.00 (0.64–1.56)
Frequency, sessions/week					
1	6	29	1.45 (0.64–3.29)	1.49 (0.65–3.42)	1.50 (0.65–3.48)
2	18	121	0.89 (0.54–1.46)	0.92 (0.56–1.53)	0.89 (0.54–1.49)
Intensity (VPA/MVPA), %					
0–30	15	98	0.88 (0.52–1.51)	0.88 (0.51–1.51)	0.85 (0.49–1.46)
> 30	9	52	1.23 (0.62–2.42)	1.42 (0.71–2.83)	1.42 (0.71–2.87)
Total					
Regularly active	247	2153	1 [Reference]	1 [Reference]	1 [Reference]
Weekend warrior	25	155	1.15 (0.76–1.73)	1.08 (0.71–1.65)	1.09 (0.71–1.65)
Frequency, sessions/week					
1	8	37	1.93 (0.95–3.92)	1.96 (0.95–4.04)	2.04 (0.98–4.24)
2	17	118	0.96 (0.59–1.57)	0.90 (0.54–1.48)	0.89 (0.54–1.46)
Intensity (VPA/MVPA), %					
0–30	19	106	1.15 (0.72–1.83)	1.06 (0.66–1.70)	1.04 (0.65–1.68)
> 30	6	49	1.14 (0.51–2.56)	1.17 (0.52–2.64)	1.23 (0.54–2.79)

*VPA* vigorous physical activity, *MVPA* moderate-to-vigorous physical activity

^a^Model 1 Adjusted for age, gender, race and ethnicity.

^b^Model 2 Adjusted for Model 1 and plus education, income, marital status, smoking, alcohol intake, HEI-2015, high blood cholesterol, family diabetes, hypertension.

^**c**^Model 3 Adjusted for Model 2 and BMI.

### Sensitivity analysis

In the sensitivity analyses, when excluding individuals who engaged in ≥ 600 min/week of MVPA and who died within the first 24 months of follow-up, similar results could be observed. That is, there were no differences in all-cause mortality risk between weekend warrior and regularly active participants, regardless of leisure-time and occupational PA, or frequency and intensity of PA (Additional file 1: Tables S9–S10).

## Discussion

This study included 6067 adults living with type 2 diabetes, and explored the association of leisure-time and occupational PA patterns with all-cause mortality. Participants who engaged in weekend warrior or regularly active PA patterns, regardless of leisure-time, occupational or total activity, experienced approximately 40%–50% reduced risk of all-cause mortality compared with those in physically inactive pattern. By contrast, there was no difference in all-cause mortality between weekend warrior and regularly active participants, suggesting that concentrating the recommended MVPA into fewer sessions or distributing it over more sessions across the week may have similar effects on lowering the risk of all-cause mortality in adults living with type 2 diabetes. Therefore, the weekend warrior PA pattern may be sufficient to reduce all-cause mortality for adults living with type 2 diabetes.

The results of this study were consistent with some previous studies conducted in middle-aged and elderly people [9, 22, 23]. A meta-analysis demonstrated that both weekend warrior and regularly active PA patterns decreased all-cause mortality risk by approximately 20% when compared with inactive pattern among the general population [9]. This study found that individuals living with type 2 diabetes engaging in the weekend warrior and regularly active PA patterns during leisure time had approximately 50% lower all-cause mortality rates compared with inactive individuals. This suggested that, compared with the inactive pattern, both the weekend warrior and regularly active PA patterns may have much better effects on reducing all-cause mortality rates in adults living with type 2 diabetes than in the general population. Therefore, organizations of public health and policy makers should prioritize efforts to encourage inactive patients living with type 2 diabetes to achieve the minimum recommended MVPA, which may yield superior health outcomes. However, two prospective cohort studies from the UK and China showed that engaging in the recommended leisure-time PA levels did not show a clear association with reduced all-cause mortality in adults living with type 2 diabetes [7]. The difference could be due to variations in study populations, classification of PA levels, and the definition of leisure-time PA. More extensive studies across diverse cultures and countries are required to interpret this conflict. Notably, no difference was observed for all-cause mortality between weekend warrior and regularly active patterns in our study, which was similar to the findings from the US National Health Interview Survey (NHIS) [22, 23]. This demonstrated that participants experienced similar lower all-cause mortality risk whether they engaged in weekend warrior or regularly active PA patterns. Therefore, the minimum recommended levels of PA may be sufficient enough in reducing mortality rate among patients living with type 2 diabetes. It’s probably because type 2 diabetes is conventionally associated with macrovascular (such as coronary heart disease and peripheral arterial disease), as well as microvascular (such as diabetic kidney disease and retinopathy) complications [24], which can be greatly improved by PA, especially MVPA [25, 26]. Although the weekend warrior and regularly active PA patterns showed an association with reduced CVD and/or cancer mortality risk among participants living with type 2 diabetes in this study, it's important to note that drawing conclusions was challenging due to the limited number of deaths observed in the weekend warrior pattern.

However, above research mainly focused on leisure-time PA because of widespread computerization and mechanization during work and chore-related activities. There is limited empirical evidence on whether similar health benefits could be observed in different types of PA. Weekend warrior and regularly active patterns of occupational PA were associated with lower risk for all-cause mortality in this study, which was in line with previous investigation from the general population [27]. Findings from the Tromsø Study indicated that, compared with low occupational PA, high and very high occupational PA decreased all-cause mortality by 20%–30% [27]. Specifically, in this study, two active patterns lowered all-cause mortality of 30% to 45% than inactive pattern. This may be speculated that individuals living with type 2 diabetes in physically inactive group engaged in less MVPA than the general population, which make the comparison more significantly. Moreover, a study from Chinese showed that in the least education group, higher occupational PA was associated with lower all-cause mortality, while this association was negative in the most educated group [28]. This implied that when examining the association of weekend warrior and other PA patterns with mortality risk, subgroup analysis according to multiple socio-demographic factors should be considered. On the contrary, results from UK Biobank indicated that there was no association between occupational PA and all-cause mortality [29]. This discrepancy may be resulted from different definition of occupational PA, classification criteria of PA levels, and study population. Further prospective studies and randomized control trials in larger samples are required to explore the association between weekend warrior activity and mortality in different types of PA among adults living with type 2 diabetes.

Results of the NHIS also suggested that longer session duration of regularly active pattern remained associated with lower all-cause mortality when compared with inactive pattern [22], which was consistent with the result in this study. In the present study, of leisure-time and occupational PA, regularly active individuals had lower all-cause mortality in longer duration of sessions compared with inactive individuals. This suggested people living with type 2 diabetes to participate in longer time of PA every session. More importantly, regardless of comparing intensity of weekend warrior with inactive or regularly active patterns, differences were not observed, which was also in line with results from the NHIS [22]. A meta-analysis also found that higher PA of any intensity was associated with lower all-cause mortality [30]. This may prompt adults living with type 2 diabetes who are unable to engage in excessive VPA to engage in the weekend warrior PA pattern, irrespective of intensity. Nevertheless, when PA was measured using wearable devices, it showed that higher-intensity PA was linked to reduced all-cause mortality [31], which contrasted the findings when comparing weekend warriors with inactive individuals, but aligned with the results of comparing regularly active individuals with inactive ones. The difference can be attributed to different assessment methods of PA, definition of intensity, and study population. It’s probably because weekend warrior participants living with type 2 diabetes mainly engaged in relatively short time of light or moderate PA, with preexisting or conditions diseases, which was similar to inactive participants. Whereas individuals in regularly active group who enjoyed PA tended to do more MPA or VPA, with better healthy conditions. Different adjustment of confounders may also contribute to conflict between our study with previous studies.

### Strengths and limitations

There are several limitations of this study. Firstly, the utilization of questionnaires for assessing PA may introduce measurement errors, in contrast to more precise methods such as accelerometers. While questionnaires are cost-effective and allow for large-scale data collection, their inherent limitations should be acknowledged. Secondly, this study only obtained a single measurement of PA at baseline, lacking repeated measurements which would provide stronger evidence of the association between PA patterns and mortality. Furthermore, the sample size of this study was relatively small, thus limiting the ability to provide robust evidence for the association of PA patterns with CVD and cancer mortality. Lastly, the potential influence of residual confounding, such as comorbidity, was not fully considered.

However, the study’s strengths include its analysis of a representative sample of US adults living with type 2 diabetes over an average follow-up of 6 years. Future cohort studies should consider combining self-reported and device-measured PA and use repeated measures of PA to reduce measurement errors and enhance the strength of association with all-cause and cause-specific mortality.

## Conclusion

The results of this extensive prospective cohort study emphasized that the weekend warrior PA pattern may have similar effects on lowering all-cause mortality as regularly active pattern among adults living with type 2 diabetes, regardless of leisure-time or occupational activity. The weekend warrior PA pattern may be sufficient to reduce all-cause mortality for adults living with type 2 diabetes. Our findings encourage inactive adults living with type 2 diabetes to aim for the minimum recommended MVPA (150 min/week). This goal could be attained through a combination of daily leisure-time and occupational activities. These results are especially pertinent for individuals living with type 2 diabetes who may experience muscle weakness and exercise intolerance, potentially limiting their ability to engage in regular physical activity during the work week.

## Supplementary Information

**Additional file 1.**

## Data Availability

The datasets generated and/or analyzed during the current study are available in the: https://wwwn.cdc.gov/nchs/nhanes/Default.aspx. Access to the data is permitted openly.

## Abbreviations

PA: Physical activity
MPA: Moderate-intensity physical activity
VPA: Vigorous-intensity physical activity
MVPA: Noderate-to-vigorous physical activity
CVD: Cardiovascular disease
NHANES: National Health and Nutrition Examination Survey
NCHS: National Center for Health Statistics
PIR: Poverty income ratio
HEI-2015: Healthy eating index-2015
BMI: Body mass index
HR: Hazard ratio
NHIS: National Health Interview Survey
