# Supplementary material for: Association between weekend warrior physical activity pattern and all-cause mortality among adults living with type 2 diabetes: a prospective cohort study from NHANES 2007 to 2018
Source: Diabetol Metab Syndr. 2024 Sep 12;16:226. doi: 10.1186/s13098-024-01455-0 (PMC11391736; doi:10.1186/s13098-024-01455-0)
Supplement: Supplementary file 1 — Additional file 1. [file 13098_2024_1455_MOESM1_ESM.docx]

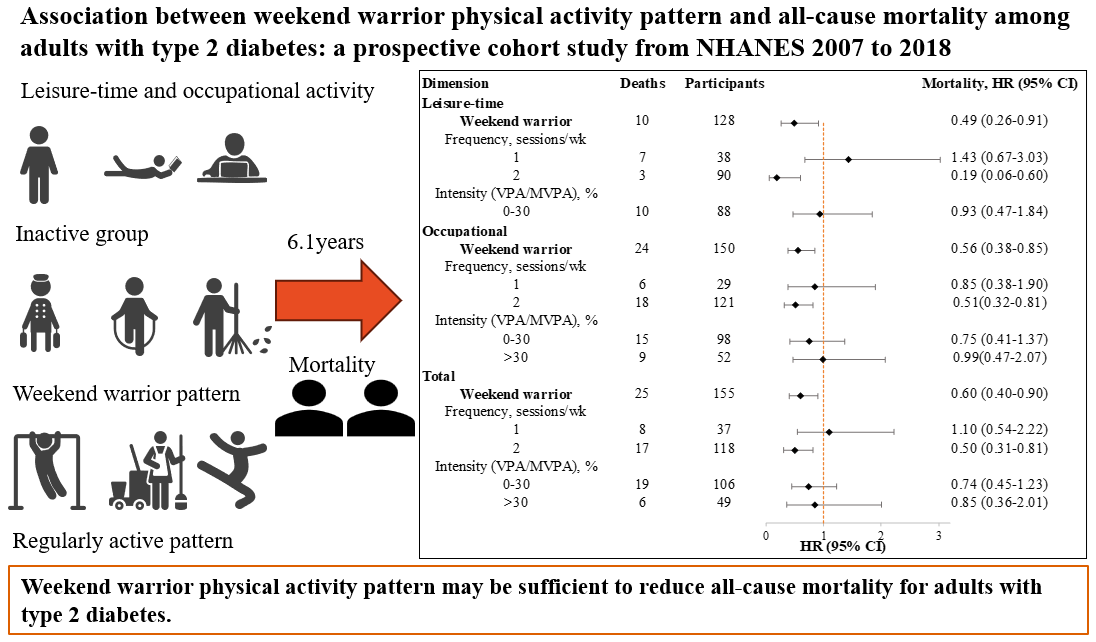


**Fig S1** Graphical abstract


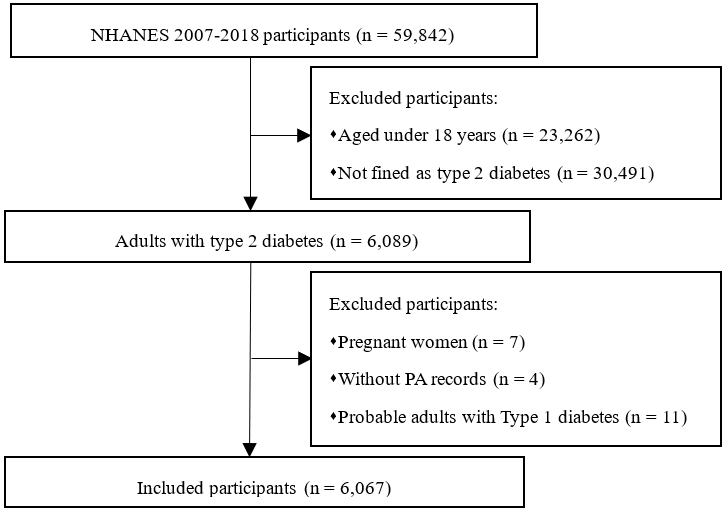


**Fig S2** Flowchart of the target population

**Table S1** Questions of leisure-time and occupational physical activity

| **No.** | **Physical Activity Questions** |
| --- | --- |
| 1 | In a typical week, on how many days do you do moderate intensity sports, fitness or recreational activities at least 10 minutes continuously? |
| 2 | How much time do you spend doing moderate intensity sports, fitness or recreational activities on a typical day? |
| 3 | In a typical week, on how many days do you do vigorous intensity sports, fitness or recreational activities at least 10 minutes continuously? |
| 4 | How much time do you spend doing vigorous intensity sports, fitness or recreational activities on a typical day? |
| 5 | In a typical week, on how many days do you do moderate intensity activities as part of your work? |
| 6 | How much time do you spend doing moderate intensity activities at work on a typical day? |
| 7 | In a typical week, on how many days do you do vigorous intensity activities as part of your work at least 10 minutes continuously? |
| 8 | How much time do you spend doing vigorous intensity activities at work on a typical day at least 10 minutes continuously? |

**Methods-S1:**

For example: 50 minutes of leisure-time MPA on a typical day, for 5 days in a typical week; 40 minutes of leisure-time VPA on a typical day, for 4 days in a typical week; 30 minutes of occupational MPA on a typical day, for 3 days in a typical week; 20 minutes of occupational VPA on a typical day, for 2 days in a typical week.

The quantification of leisure-time MVPA = 50 × 5 + 40 × 4 × 2 = 570 min/wk

The quantification of occupational MVPA = 30 × 3 + 20 × 2 × 2 = 170 min/wk

The amount of total MVPA = 570 + 170 = 740 min/wk

**Methods-S2:**

For instance: a participant who engaged in 20 min/wk of leisure-time VPA and 60 min/wk of leisure-time MPA accumulated a total of 100 min/wk of leisure-time MVPA, with VPA accounting for 40.0% of MVPA (VPA proportion calculated as 20 × 2 / ([20 × 2] + 60).

**Table S2** The distribution of missing variables

| **Variable name** | **Missing data (No.)** | **Missing data (%)** |
| --- | --- | --- |
| Age | 0 | 0 |
| Gender | 0 | 0 |
| Race | 0 | 0 |
| Education level | 14 | 0.23 |
| Poverty income ratio | 684 | 11.27 |
| Marital status | 19 | 0.31 |
| BMI | 311 | 5.13 |
| Cigarette smoking status | 51 | 0.84 |
| Alcohol intake | 369 | 6.08 |
| HEI-2015 | 673 | 11.09 |
| High blood cholesterol | 146 | 2.40 |
| Family Diabetes | 174 | 2.87 |
| Hypertension | 14 | 0.23 |

Abbreviation: BMI, body mass index; HEI-2015, healthy eating index-2015.

**Table S3** Baseline characteristics of participants in occupational PA pattern

| **Variable** | **Occupational physical activity pattern, No. %** | | |  |
| --- | --- | --- | --- | --- |
|  | **Inactive** | **Weekend warrior** | **Regularly active** | **Overall** |
| Total | 4,570 (75.3) | 150 (2.5) | 1,347 (22.2) | 6,067 (100.0) |
| Age |  |  |  |  |
| 18-44 | 478 (10.5) | 15 (10.0) | 252 (18.7) | 745 (12.3) |
| 45-64 | 1,906 (41.7) | 58 (38.7) | 654 (48.6) | 2,618 (43.2) |
| 65-84 | 2,186 (47.8) | 77 (51.3) | 441 (32.7) | 2,704 (44.6) |
| Gender |  |  |  |  |
| Male | 2,229 (48.8) | 94 (62.7) | 833 (61.8) | 3,156 (52.0) |
| Female | 2,341 (51.2) | 56 (37.3) | 514 (38.2) | 2,911 (48.0) |
| Race and ethnicity |  |  |  |  |
| Hispanic | 1,325 (30.0) | 31 (20.7) | 384 (28.5) | 1,740 (28.7) |
| Non-Hispanic White | 1,430 (31.3) | 62 (41.3) | 528 (39.2) | 2,020 (33.3) |
| Non-Hispanic Black | 1,247 (27.3) | 43 (28.7) | 310 (23.0) | 1,600 (26.4) |
| Other | 568 (12.4) | 14 (9.3) | 125 (9.3) | 707 (11.7) |
| Education level |  |  |  |  |
| <High school degree | 1,696 (37.1) | 45 (30.0) | 371 (27.5) | 2,112 (34.8) |
| High school degree | 998 (21.8) | 32 (21.3) | 367 (27.2) | 1,397 (23.0) |
| >High school degree | 1,876 (41.1) | 73 (48.7) | 609 (45.2) | 2,558 (42.2) |
| Poverty income ratio |  |  |  |  |
| <1 | 1,033 (22.6) | 29 (19.3) | 255 (18.9) | 1,317 (21.7) |
| [1,3) | 2,381 (52.1) | 73 (48.7) | 719 (53.4) | 3,173 (52.3) |
| >=3 | 1,156 (25.3) | 48 (32.0) | 373 (27.7) | 1,577 (26.0) |
| Marital status |  |  |  |  |
| Married/living with partner | 2,607 (57) | 94 (62.7) | 858 (63.7) | 3,559 (58.7) |
| Widowed/divorced/ separated | 1,526 (33.4) | 47 (31.3) | 343 (25.5) | 1,916 (31.6) |
| Never married | 437 (9.6) | 9 (6.0) | 146 (10.8) | 592 (9.8) |
| BMI |  |  |  |  |
| ≤18.4 | 19 (0.4) | 1 (0.7) | 2 (0.1) | 22 (0.4) |
| 18.5-24.9 | 592 (13) | 15 (10.0) | 141 (10.5) | 748 (12.3) |
| 25-29.9 | 1,247 (27.3) | 42 (28.0) | 356 (26.4) | 1,645 (27.1) |
| ≥30 | 2,712 (59.3) | 92 (61.3) | 848 (63.0) | 3,652 (60.2) |
| Cigarette smoking status |  |  |  |  |
| Never | 2,394 (52.4) | 61 (40.7) | 625 (46.4) | 3,080 (50.8) |
| Former | 1,590 (34.8) | 74 (49.3) | 515 (38.2) | 2,179 (35.9) |
| Current | 586 (12.8) | 15 (10.0) | 207 (15.4) | 808 (13.3) |
| Alcohol intake |  |  |  |  |
| Never | 2,135 (46.7) | 58 (38.7) | 519 (38.5) | 2,712 (44.7) |
| Former | 1,217 (26.6) | 34 (22.7) | 298 (22.1) | 1,549 (25.5) |
| Current | 1,218 (26.7) | 58 (38.7) | 530 (39.3) | 1,806 (29.8) |
| HEI-2015 |  |  |  |  |
| T1 | 1,431 (31.3) | 49 (32.7) | 543 (40.3) | 2,023 (33.3) |
| T2 | 1,590 (34.8) | 49 (32.7) | 383 (28.4) | 2,022 (33.3) |
| T3 | 1,549 (33.9) | 52 (34.7) | 421 (31.3) | 2,022 (33.3) |
| High blood cholesterol |  |  |  |  |
| Yes | 2,705 (59.2) | 87 (58.0) | 785 (58.3) | 3,577 (59.0) |
| No | 1,865 (40.8) | 63 (42.0) | 562 (41.7) | 2,490 (41.0) |
| Family Diabetes |  |  |  |  |
| Yes | 3,006 (65.8) | 92 (61.3) | 930 (69.0) | 4,028 (66.4) |
| No | 1,564 (34.2) | 58 (38.7) | 417 (31.0) | 2,039 (33.6) |
| Hypertension |  |  |  |  |
| Yes | 3,082 (67.4) | 97 (64.7) | 846 (62.8) | 4,025 (66.3) |
| No | 1,488 (32.6) | 53 (35.3) | 501 (37.2) | 2,042 (33.7) |

Abbreviation: BMI, body mass index; HEI-2015, healthy eating index-2015.


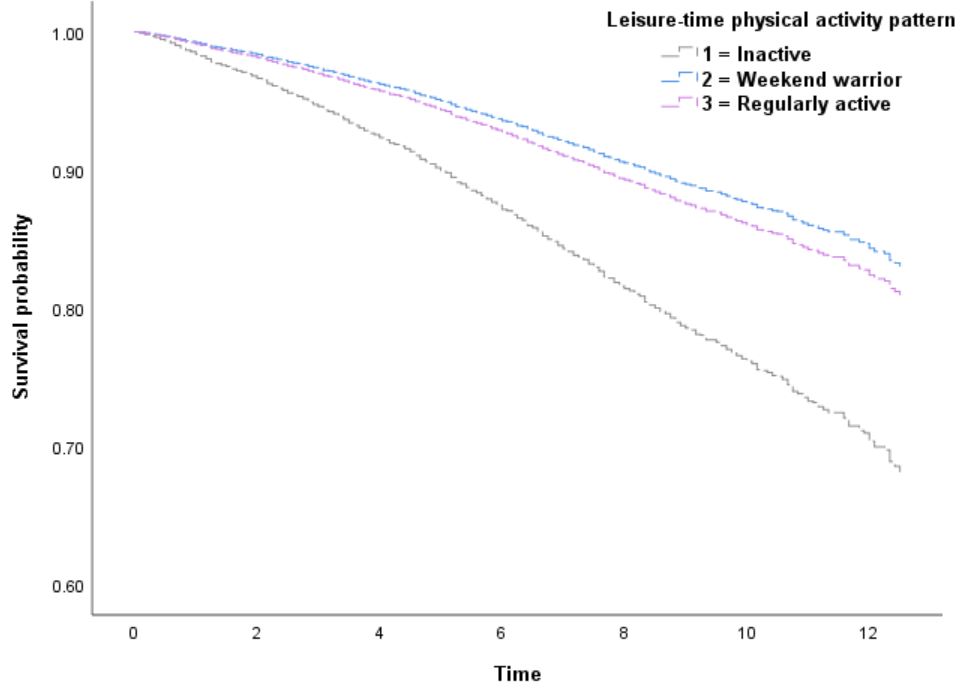


**Fig S3** Kaplan–Meier curves for all-cause mortality in leisure-time PA pattern


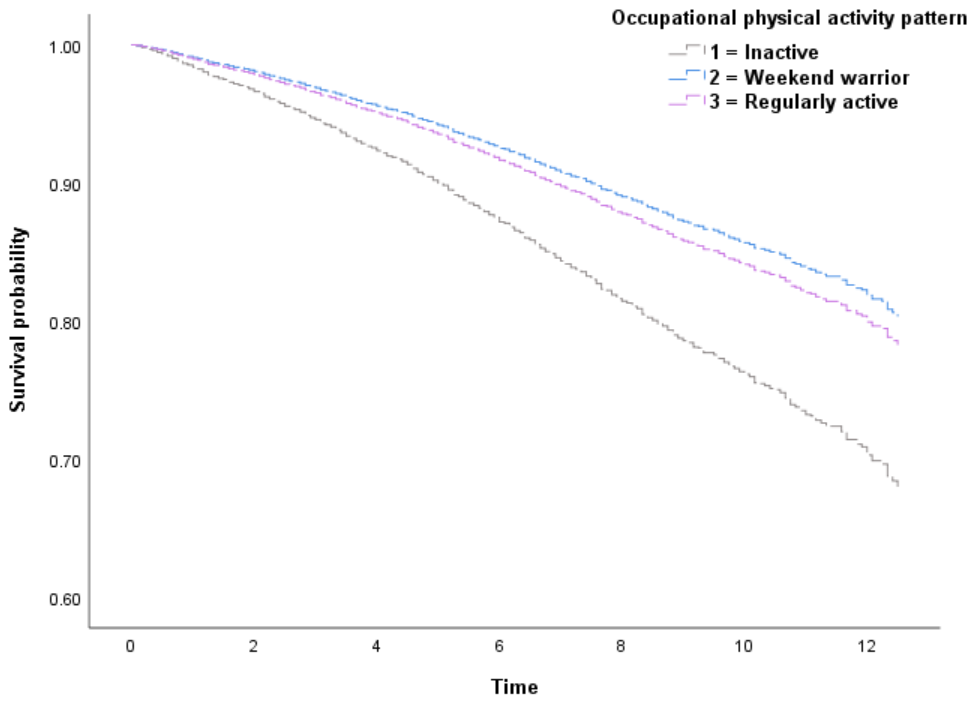


**Fig S4** Kaplan–Meier curves for all-cause mortality in occupational PA pattern

**Table S4** Association between PA pattern and cause-specific mortality

| **Dimension** | **Outcomes** | **Deaths** | **Participants** | **Mortality, HR (95% CI)** | | |
| --- | --- | --- | --- | --- | --- | --- |
|  |  |  |  | **Model 1^#^** | **Model 2*** | **Model 3^** |
| Leisure-time | CVD mortality |  |  |  |  |  |
|  | Inactive | 304 | 4,885 | 1 [Reference] | 1 [Reference] | 1 [Reference] |
|  | Weekend warrior | 3 | 128 | 0.41 (0.13-1.27) | 0.56 (0.18-1.75) | 0.57 (0.18-1.78) |
|  | Regularly active | 28 | 1,054 | 0.46 (0.31-0.67) | 0.53 (0.36-0.79) | 0.53 (0.36-0.79) |
|  | Cancer mortality |  |  |  |  |  |
|  | Inactive | 198 | 4,885 | 1 [Reference] | 1 [Reference] | 1 [Reference] |
|  | Weekend warrior | 3 | 128 | 0.61 (0.19-1.90) | 0.78 (0.25-2.45) | 0.78 (0.25-2.45) |
|  | Regularly active | 230 | 1,054 | 0.70 (0.47-1.04) | 0.81 (0.54-1.21) | 0.81 (0.54-1.20) |
| Occupational | CVD mortality |  |  |  |  |  |
|  | Inactive | 287 | 4,570 | 1 [Reference] | 1 [Reference] | 1 [Reference] |
|  | Weekend warrior | 7 | 150 | 0.53 (0.25-1.31) | 0.6190.29-1.30） | 0.62 (0.29-1.31) |
|  | Regularly active | 41 | 1,347 | 0.55 (0.39-0.76) | 0.61（0.44-0.85） | 0.61 (0.44-0.86) |
|  | Cancer mortality |  |  |  |  |  |
|  | Inactive | 193 | 4,570 | 1 [Reference] | 1 [Reference] | 1 [Reference] |
|  | Weekend warrior | 8 | 150 | 0.89 (0.44-1.82) | 0.96 (0.47-1.97) | 0.97 (0.47-1.98) |
|  | Regularly active | 29 | 1,347 | 0.57 (0.39-0.85) | 0.61 (0.41-0.91) | 0.61 (0.41-0.91) |

Abbreviations: PA, physical activity; CVD, cardiovascular disease.

^#^Model 1 Adjusted for age, gender, race and ethnicity.

*Model 2 Adjusted for Model 1 and plus education, income, marital status, smoking, alcohol intake, HEI-2015, high blood cholesterol, family diabetes, hypertension.

^Model 3 Adjusted for Model 2 and BMI.

**Table S****5** Association between weekend warrior PA pattern and all-cause mortality, by frequency and intensity of activity

| **Dimension** | **Deaths** | **Participants** | **Mortality, HR (95% CI)** | | |
| --- | --- | --- | --- | --- | --- |
|  |  |  | **Model 1^#^** | **Model 2*** | **Model 3^** |
| Leisure-time | | | | | |
| Inactive | 1,087 | 4,885 | 1 [Reference] | 1 [Reference] | 1 [Reference] |
| Weekend warrior | 10 | 128 | 0.38 (0.20-0.70) | 0.49 (0.26-0.91) | 0.49 (0.26-0.91) |
| Frequency, sessions/wk |  |  |  |  |  |
| 1 | 7 | 38 | 0.96 (0.45-2.01) | 1.44 (0.69-3.05) | 1.43 (0.67-3.03) |
| 2 | 3 | 90 | 0.16 (0.05-0.48) | 0.19 (0.06-0.60) | 0.19 (0.06-0.60) |
| Intensity (VPA/MVPA), % |  |  |  |  |  |
| 0-30 | 10 | 88 | 0.74 (0.39-1.44) | 0.97 (0.49-1.92) | 0.93 (0.47-1.84) |
| >30 | 0 | 40 | NA | NA | NA |
| Occupational | | | | | |
| Inactive | 1,029 | 4,570 | 1 [Reference] | 1 [Reference] | 1 [Reference] |
| Weekend warrior | 24 | 150 | 0.52 (0.35-0.78) | 0.56 (0.37-0.84) | 0.56 (0.38-0.85) |
| Frequency, sessions/wk |  |  |  |  |  |
| 1 | 6 | 29 | 0.77 (0.34-1.71) | 0.82 (0.37-1.83) | 0.85 (0.38-1.90) |
| 2 | 18 | 121 | 0.47 (0.30-0.75) | 0.51 (0.32-0.81) | 0.51 (0.32-0.81) |
| Intensity (VPA/MVPA), % |  |  |  |  |  |
| 0-30 | 15 | 98 | 0.75 (0.42-1.32) | 0.71 (0.39-1.28) | 0.75 (0.41-1.37) |
| >30 | 9 | 52 | 1.01 (0.50-2.04) | 1.06 (0.51-2.17) | 0.99 (0.47-2.07) |
| Total |  |  |  |  |  |
| Inactive | 934 | 3,759 | 1 [Reference] | 1 [Reference] | 1 [Reference] |
| Weekend warrior | 25 | 155 | 0.56 (0.38-0.84) | 0.60 (0.41-0.90) | 0.60 (0.40-0.90) |
| Frequency, sessions/wk |  |  |  |  |  |
| 1 | 8 | 37 | 0.90 (0.45-1.80) | 1.09 (0.54-2.19) | 1.10 (0.54-2.22) |
| 2 | 17 | 118 | 0.48 (0.30-0.78) | 0.50 (0.31-0.81) | 0.50 (0.31-0.81) |
| Intensity (VPA/MVPA), % |  |  |  |  |  |
| 0-30 | 19 | 106 | 0.85 (0.52-1.39) | 0.78 (0.47-1.28) | 0.74 (0.45-1.23) |
| >30 | 6 | 49 | 0.95 (0.41-2.16) | 0.92 (0.39-2.15) | 0.85 (0.36-2.01) |

Abbreviation: VPA, vigorous physical activity; MVPA, moderate-to-vigorous physical activity

^#^Model 1 Adjusted for age, gender, race and ethnicity.

*Model 2 Adjusted for Model 1 and plus education, income, marital status, smoking, alcohol intake, HEI-2015, high blood cholesterol, family diabetes, hypertension.

^Model 3 Adjusted for Model 2 and BMI.

**Table S6** Association between regularly active PA pattern and all-cause mortality, by frequency, duration, and intensity of activity

| **Dimension** | **Deaths** | **Participants** | **Mortality, HR (95% CI)** | | |
| --- | --- | --- | --- | --- | --- |
|  |  |  | **Model 1^#^** | **Model 2*** | **Model 3^** |
| Leisure-time | | | | | |
| Inactive | 1,087 | 4,885 | 1 [Reference] | 1 [Reference] | 1 [Reference] |
| Regularly active | 109 | 1,054 | 0.49 (0.40-0.60) | 0.56（0.46-0.68） | 0.55（0.45-0.68） |
| Frequency, sessions/wk |  |  |  |  |  |
| 3-4 | 33 | 377 | 0.44 (0.31-0.63) | 0.53 (0.37-0.75) | 0.54 (0.38-0.77) |
| ≥5 | 76 | 677 | 0.52 (0.41-0.65) | 0.57 (0.45-0.72) | 0.56 (0.44-0.70) |
| Duration of session, min |  |  |  |  |  |
| 0-30 | 21 | 137 | 0.75 (0.49-1.16) | 0.75 (0.48-1.15) | 0.72 (0.47-1.12) |
| >30 | 88 | 917 | 0.46 (0.37-0.57) | 0.52 (0.42-0.65) | 0.52 (0.42-0.65) |
| Intensity (VPA/MVPA), % |  |  |  |  |  |
| 0-30 | 86 | 674 | 0.84 (0.63-1.13) | 0.86 (0.63-1.15) | 0.86 (0.64-1.16) |
| >30 | 23 | 380 | 0.68 (0.43-1.07) | 0.72 (0.46-1.14) | 0.71 (0.45-1.13) |
| Occupational | | | | | |
| Inactive | 1,029 | 4,570 | 1 [Reference] | 1 [Reference] | 1 [Reference] |
| Regularly active | 153 | 1,347 | 0.58 (0.49-0.68) | 0.63 (0.53-0.75） | 0.64 (0.54-0.76） |
| Frequency, sessions/wk |  |  |  |  |  |
| 3-4 | 49 | 330 | 0.74 (0.55-0.98) | 0.81 (0.61-1.08) | 0.83 (0.62-1.10) |
| ≥5 | 104 | 1,017 | 0.52 (0.43-0.64) | 0.57 (0.47-0.70) | 0.58 (0.47-0.71) |
| Duration of session, min |  |  |  |  |  |
| 0-30 | 9 | 62 | 0.51 (0.26-0.98) | 0.59 (0.31-1.15) | 0.60 (0.31-1.17) |
| >30 | 144 | 1,285 | 0.58 (0.49-0.69) | 0.63 (0.53-0.76) | 0.64 (0.54-0.76) |
| Intensity (VPA/MVPA), % |  |  |  |  |  |
| 0-30 | 103 | 712 | 0.91 (0.67-1.25) | 0.91 (0.66-1.24) | 0.96 (0.70-1.32) |
| >30 | 50 | 635 | 0.65 (0.45-0.95) | 0.60 (0.41-0.88) | 0.61 (0.42-0.90) |
| Total |  |  |  |  |  |
| Inactive | 934 | 3,759 | 1 [Reference] | 1 [Reference] | 1 [Reference] |
| Regularly active | 247 | 2,153 | 0.50 (0.43-0.58) | 0.57（0.49-0.65） | 0.57（0.49-0.65） |
| Frequency, sessions/wk |  |  |  |  |  |
| 3-4 | 59 | 435 | 0.63 (0.48-0.82) | 0.72 (0.56-0.95) | 0.73 (0.56-0.96) |
| ≥5 | 188 | 1,718 | 0.47 (0.40-0.55) | 0.53 (0.45-0.62) | 0.53 (0.45-0.62) |
| Duration of session, min |  |  |  |  |  |
| 0-30 | 29 | 181 | 0.63 (0.43-0.91) | 0.68 (0.47-0.99) | 0.68 (0.47-0.99) |
| >30 | 218 | 1,972 | 0.49 (0.42-0.57) | 0.55 (0.47-0.64) | 0.55 (0.48-0.64) |
| Intensity (VPA/MVPA), % |  |  |  |  |  |
| 0-30 | 173 | 1,224 | 0.81 (0.64-1.02) | 0.84 (0.66-1.06) | 0.85 (0.67-1.08) |
| >30 | 74 | 929 | 0.67 (0.50-0.90) | 0.68 (0.50-0.91) | 0.67 (0.50-0.91) |

Abbreviation: VPA, vigorous physical activity; MVPA, moderate-to-vigorous physical activity

^#^Model 1 Adjusted for age, gender, race and ethnicity.

*Model 2 Adjusted for Model 1 and plus education, income, marital status, smoking, alcohol intake, HEI-2015, high blood cholesterol, family diabetes, hypertension.

^Model 3 Adjusted for Model 2 and BMI.

**Table S7** Association between weekend warrior PA pattern and cause-specific mortality, with regularly active pattern as the reference group in leisure-time PA

| **Outcomes** | **Mortality, HR (95% CI)** | | |
| --- | --- | --- | --- |
|  | **Model 1^#^** | **Model 2*** | **Model 3^** |
| All-cause mortality |  |  |  |
| Regularly active | 1 [Reference] | 1 [Reference] | 1 [Reference] |
| Weekend warrior | 0.73 (0.38-1.40) | 0.83 (0.42-1.62) | 0.82 (0.42-1.61) |
| CVD mortality |  |  |  |
| Regularly active | 1 [Reference] | 1 [Reference] | 1 [Reference] |
| Weekend warrior | 0.85 (0.26-2.80) | 1.04（0.30-3.65）） | 1.06 (0.30-3.68) |
| Cancer mortality |  |  |  |
| Regularly active | 1 [Reference] | 1 [Reference] | 1 [Reference] |
| Weekend warrior | 0.89 (0.27-2.94) | 0.92 (0.25-3.33) | 0.92 (0.25-3.32) |

Abbreviations: CVD, cardiovascular disease.

^#^Model 1 Adjusted for age, gender, race and ethnicity.

*Model 2 Adjusted for Model 1 and plus education, income, marital status, smoking, alcohol intake, HEI-2015, high blood cholesterol, family diabetes, hypertension.

^Model 3 Adjusted for Model 2 and BMI.

**Table S8** Association between weekend warrior PA pattern and cause-specific mortality, with regularly active pattern as the reference group in occupational PA

| **Outcomes** | **Mortality, HR (95% CI)** | | |
| --- | --- | --- | --- |
|  | **Model 1^#^** | **Model 2*** | **Model 3^** |
| All-cause mortality |  |  |  |
| Regularly active | 1 [Reference] | 1 [Reference] | 1 [Reference] |
| Weekend warrior | 0.99 (0.64-1.53) | 1.02 (0.66-1.60) | 1.00 (0.64-1.56） |
| CVD mortality |  |  |  |
| Regularly active | 1 [Reference] | 1 [Reference] | 1 [Reference] |
| Weekend warrior | 1.09 (0.48-2.46) | 1.26 (0.54-2.91) | 1.24 (0.54-2.87) |
| Cancer mortality |  |  |  |
| Regularly active | 1 [Reference] | 1 [Reference] | 1 [Reference] |
| Weekend warrior | 1.72 (0.77-3.83) | 1.73 (0.76-3.94) | 1.72 (0.75-3.92) |

Abbreviations: CVD, cardiovascular disease.

^#^Model 1 Adjusted for age, gender, race and ethnicity.

*Model 2 Adjusted for Model 1 and plus education, income, marital status, smoking, alcohol intake, HEI-2015, high blood cholesterol, family diabetes, hypertension.

^Model 3 Adjusted for Model 2 and BMI.

**Sensitivity analysis**

**Table S9** Sensitivity analysis excluding participants with ≥600 min/wk of PA: Association between weekend warrior PA pattern and all-cause mortality, with regularly active pattern as the reference group

| **Dimension** | **Mortality, HR (95% CI)** | | |
| --- | --- | --- | --- |
|  | **Model 1^#^** | **Model 2*** | **Model 3^** |
| Leisure-time |  |  |  |
| Regularly active | 1 [Reference] | 1 [Reference] | 1 [Reference] |
| Weekend warrior | 0.71 (0.37-1.38) | 0.86 (0.43-1.70) | 0.86 (0.43-1.71) |
| Frequency, sessions/wk |  |  |  |
| 1 | 1.60 (0.73-3.50) | 2.25 (0.96-5.28) | 2.24 (0.95-5.26) |
| 2 | 0.32 (0.1-1.00) | 0.36 (0.11-1.16) | 0.36 (0.11-1.16) |
| Intensity (VPA/MVPA), % |  |  |  |
| 0-30 | 0.88 (0.46-1.70) | 1.08 (0.54-2.16) | 1.08 (0.54-2.16) |
| >30 | NA | NA | NA |
| Occupational |  |  |  |
| Regularly active | 1 [Reference] | 1 [Reference] | 1 [Reference] |
| Weekend warrior | 0.91 (0.55-1.49) | 0.86 (0.51-1.46) | 0.82 (0.48-1.40) |
| Frequency, sessions/wk |  |  |  |
| 1 | 1.51 (0.65-3.52) | 1.42 (0.58-3.47) | 1.49 (0.60-3.66) |
| 2 | 0.78 (0.44-1.38) | 0.75 (0.42-1.34) | 0.69 (0.38-1.26) |
| Intensity (VPA/MVPA), % |  |  |  |
| 0-30 | 0.76 (0.41-1.40) | 0.66 (0.35-1.24) | 0.63 (0.33-1.21) |
| >30 | 1.27 (0.62-2.60) | 1.53 (0.73-3.22) | 1.48 (0.68-3.22) |

Abbreviation: VPA, vigorous physical activity; MVPA, moderate-to-vigorous physical activity

^#^Model 1 Adjusted for age, gender, race and ethnicity.

*Model 2 Adjusted for Model 1 and plus education, income, marital status, smoking, alcohol intake, HEI-2015, high blood cholesterol, family diabetes, hypertension.

^Model 3 Adjusted for Model 2 and BMI.

**Table S10** Sensitivity analysis excluding individuals who passed away within the first 24 months of follow-up: Association between weekend warrior PA pattern and all-cause mortality, with regularly active pattern as the reference group

| **Dimension** | **Mortality, HR (95% CI)** | | |
| --- | --- | --- | --- |
|  | **Model 1^#^** | **Model 2*** | **Model 3^** |
| Leisure-time |  |  |  |
| Regularly active | 1 [Reference] | 1 [Reference] | 1 [Reference] |
| Weekend warrior | 0.71 (0.34-1.46) | 0.79 (0.37-1.67) | 0.85 (0.37-1.67) |
| Frequency, sessions/wk |  |  |  |
| 1 | 2.06 (0.89-4.78) | 2.46 (0.99-6.09) | 2.41 (0.97-5.99) |
| 2 | 0.24 (0.06-0.97) | 0.26 (0.06-1.09) | 0.26 (0.06-1.09) |
| Intensity (VPA/MVPA), % |  |  |  |
| 0-30 | 0.88 (0.42-1.81) | 0.98 (0.46-2.07) | 0.96 (0.45-2.05) |
| >30 | NA | NA | NA |
| Occupational |  |  |  |
| Regularly active | 1 [Reference] | 1 [Reference] | 1 [Reference] |
| Weekend warrior | 1.03 (0.62-1.70) | 1.04 (0.62-1.75) | 1.02 (0.60-1.72) |
| Frequency, sessions/wk |  |  |  |
| 1 | 2.01 (0.88-4.60) | 2.20 (0.94-5.12) | 2.24 (0.95-5.30) |
| 2 | 0.82 (0.45-1.50) | 0.81 (0.44-1.51) | 0.79 (0.43-1.48) |
| Intensity (VPA/MVPA), % |  |  |  |
| 0-30 | 1.13 (0.64-2.00) | 1.13 (0.63-2.01) | 1.10 (0.62-1.97) |
| >30 | 0.77 (0.28-2.10) | 0.82 (0.30-2.28) | 0.81 (0.29-2.25) |

Abbreviation: VPA, vigorous physical activity; MVPA, moderate-to-vigorous physical activity

^#^Model 1 Adjusted for age, gender, race and ethnicity.

*Model 2 Adjusted for Model 1 and plus education, income, marital status, smoking, alcohol intake, HEI-2015, high blood cholesterol, family diabetes, hypertension.

^Model 3 Adjusted for Model 2 and BMI.
